# The David Versus the Bentall Procedure for Acute Type A Aortic Dissection

**DOI:** 10.3390/jcdd11110370

**Published:** 2024-11-19

**Authors:** Fausto Biancari, Giorgio Mastroiacovo, Mauro Rinaldi, Luisa Ferrante, Timo Mäkikallio, Tatu Juvonen, Giovanni Mariscalco, Zein El-Dean, Matteo Pettinari, Javier Rodriguez Lega, Angel G. Pinto, Andrea Perrotti, Francesco Onorati, Konrad Wisniewski, Till Demal, Petr Kacer, Jan Rocek, Dario Di Perna, Igor Vendramin, Daniela Piani, Eduard Quintana, Robert Pruna-Guillen, Joscha Buech, Caroline Radner, Manoj Kuduvalli, Amer Harky, Antonio Fiore, Angelo M. Dell’Aquila, Giuseppe Gatti, Lenard Conradi, Mark Field, Arianna Galotta, Daniele Fileccia, Giuseppe Nanci, Sven Peterss

**Affiliations:** 1 Department of Cardiovascular Surgery, Centro Cardiologico Monzino IRCCS, 20138 Milan, Italy; 2 Cardiac Surgery, Molinette Hospital, University of Turin, 10126 Turin, Italy; 3 Department of Medicine, South-Karelia Central Hospital, University of Helsinki, 53130 Lappeenranta, Finland; 4 Heart and Lung Center, Helsinki University Hospital, University of Helsinki, 00029 Helsinki, Finland; 5 Faculty of Medicine, University of Oulu, 90220 Oulu, Finland; 6 Department of Cardiac Surgery, Glenfield Hospital, Leicester LE3 9QP, UK; 7 Department of Cardiac Surgery, Saint Luc Hospital, 1200 Louvain, Belgium; 8 Cardiovascular Surgery Department, University Hospital Gregorio Marañón, 28007 Madrid, Spain; 9 Department of Thoracic and Cardiovascular Surgery, University of Franche-Comte, 25000 Besancon, France; 10 Division of Cardiac Surgery, University of Verona Medical School, 37134 Verona, Italy; 11 Department of Cardiothoracic Surgery, University Hospital Muenster, 48149 Muenster, Germany; 12 Department of Cardiovascular Surgery, University Heart and Vascular Center of Hamburg, 20251 Hamburg, Germany; 13 Department of Cardiac Surgery, University Hospital Kralovske Vinohrady, Charles University, 10000 Prague, Czech Republic; 14 Department of Cardiac Surgery, Centre Hospitalier Annecy Genevois, 74370 Epagny Metz-Tessy, France; 15 Cardiothoracic Department, Azienda Sanitaria Universitaria Friuli Centrale, 33100 Udine, Italy; 16 Department of Cardiovascular Surgery, Hospital Clínic de Barcelona, University of Barcelona, 08036 Barcelona, Spain; 17 Department of Cardiac Surgery, LMU University Hospital, Ludwig Maximilian University, 80336 Munich, Germany; 18 German Centre for Cardiovascular Research, Partner Site Munich Heart Alliance, 80336 Munich, Germany; 19 Liverpool Heart and Chest Hospital, Liverpool L14 3PE, UK; 20 Department of Cardiac Surgery, Hôpitaux Universitaires Henri Mondor, Assistance Publique-Hôpitaux de Paris, 94000 Créteil, France; antonio.fiore@aphp.fr; 21 Université Paris Est Créteil, Inserm, IMRB U955, CEpiA Team, 94000 Créteil, France; 22 Department of Cardiac Surgery, Martin Luther University Halle-Wittenberg, 06108 Halle, Germany; 23 Division of Cardiac Surgery, Cardio-Thoracic and Vascular Department, Azienda Sanitaria Universitaria Giuliano Isontina, 34148 Trieste, Italy; 24 Department of Cardiac Surgery, Cologne University Hospital, 50937 Cologne, Germany; 25 Unit of Biostatistics, Centro Cardiologico Monzino IRCCS, 20138 Milan, Italy

**Keywords:** type A aortic dissection, aortic root, David procedure, Bentall procedure

## Abstract

**Background**: Type A aortic dissection (TAAD) is a life-threatening condition which requires prompt diagnosis and surgical treatment. When TAAD involves the aortic root, aortic valve-sparing or Bentall procedures are the main surgical treatment options. **Method:** The subjects of this analysis were 3735 patients included in the European Registry of Type A Aortic Dissection (ERTAAD). Propensity score matching was performed by estimating a propensity score from being treated with the Bentall or the David procedure using multilevel mixed-effects logistics, considering the cluster effect of the participating hospitals. **Results:** A Bentall procedure was performed in 862 patients, while a David operation was performed in 139 patients. The proportion of aortic root replacement, as well as the different techniques of aortic root replacement, varied significantly between the participating hospitals (*p* < 0.001). After propensity score matching, we obtained two groups of 115 patients each, and no statistical differences were reported in terms of postoperative outcomes, except for the rate of dialysis, which was higher in the patients requiring a Bentall procedure (17.4% vs. 7.0%, *p*-value 0.016). In the unmatched cohorts, the David procedure was associated with a lower 10-year mortality rate compared to the Bentall procedure (30.1% vs. 45.6%, *p*-value 0.004), but no difference was observed after matching (30.0% vs. 43.9%, *p*-value 0.082). After 10 years, no differences were observed in terms of proximal aortic reoperation (3.9% vs. 4.1%, *p*-value 0.954), even after propensity score matching (2.8% vs. 1.8%, *p*-value 0.994). **Conclusions:** The David and Bentall procedures are durable treatment methods for TAAD. When feasible, it is advisable that the David procedure is performed for acute TAAD by surgeons with experience with this demanding surgical technique.

## 1. Introduction

Type A aortic dissection (TAAD) is a life-threatening condition which requires prompt diagnosis and surgical treatment [1,2]. When the aortic root is involved and severely injured by the dissection and/or dilated, the Bentall and David or Yacoub procedures are the most common procedures for aortic root replacement.

Arabkhani et al. [3] demonstrated that aortic root replacement surgery is characterized by a better long-term survival and a lower rate of reinterventions compared to a conservative surgical approach. Therefore, aggressive root replacement is safe and can be applied in TAADs with good long-term clinical results and without increased hospital mortality. When TAAD involves the aortic root, the evaluation of the structural and functional status of the aortic valve is of relevance to decide whether to perform a valve-sparing aortic root replacement or a Bentall operation [4]. When the aortic valve has no structural alterations, the aortic valve reimplantation technique, that is, the David procedure, is an attractive but technically more demanding procedure than the Bentall procedure [5]. The present study aimed to investigate whether there were significant differences between the two surgical techniques in terms of early postoperative adverse events and long-term durability in a multicenter series of patients operated on for acute TAAD.

## 2. Patients and Methods

### 2.1. Study Population

The European Registry of Type A Aortic Dissection (ERTAAD) was a retrospective multicenter study including data on 3735 consecutive patients who underwent aortic surgery for acute TAAD at 17 centers for cardiac surgery in eight European countries (Belgium, Czech Republic, Finland, France, Germany, Italy, Spain, and the United Kingdom) from January 2005 to March 2021. The study was approved by the Ethical Review Board of the Helsinki University Central Hospital, Finland (21 April 2021, diary no. HUS/237/2021) and by the Review Board of each participating hospital. The requirement for informed consent was waived because of the retrospective nature of the registry.

Patients with onset of symptoms related to TAAD within 7 days before the operation were included in this registry. The other inclusion and exclusion criteria of the registry and the definition criteria for clinical, operative, and outcome variables have been previously reported [6]. For the present study, only patients who underwent aortic root replacement using the David reimplantation technique or the Bentall technique were included in this analysis. Patients who underwent the Yacoub procedure were excluded from this analysis because of the small sample size.

### 2.2. Study Outcomes

The primary outcomes of this analysis were long-term mortality and a repeat procedure on the aortic root and/or aortic valve. The secondary outcomes were in-hospital mortality, stroke, paraplegia or paraparesis, tetraplegia or tetraparesis, mesenteric ischemia, sepsis, dialysis, reintervention for intrathoracic bleeding, mediastinitis, heart failure, need for mechanical circulatory support, implantation of venoarterial extracorporeal membrane oxygenation, and surgery for intestinal complications.

### 2.3. Statistical Analysis

Continuous variables were reported as means and standard deviations, while categorical variables were reported as counts and percentages. The Mann–Whitney test was used to compare continuous variables between the study groups. The Chi-square and Fisher’s exact tests were used to analyze differences in categorical variables. Propensity score matching was performed by estimating a propensity score for being treated with the Bentall or David procedure using multilevel mixed-effects logistics, considering the cluster effect of the participating hospitals. Proximal aortic reoperation might have been hindered by patient death, and a competing risk analysis was performed considering all-cause death as the competing event. The following baseline and operative variables were included as covariates in the regression model to calculate the propensity score: age, gender, genetic aortic syndromes, moderate-to-severe aortic valve insufficiency, bicuspid aortic valve, iatrogenic TAAD, diabetes, stroke, pulmonary disease, extracardiac arteriopathy, prior cardiac surgery, preoperative cardiac massage, cardiogenic shock requiring inotropes, invasive mechanical ventilation, cerebral malperfusion, spinal malperfusion, renal malperfusion, mesenteric malperfusion, peripheral malperfusion, salvage procedure, partial or total aortic arch repair, and concomitant coronary artery bypass grafting. Matching was performed using a caliper width of 0.05. Standardized difference (SD) < 0.10 indicated balanced variables between the matched study cohorts. The risk estimate was reported as a subdistributional hazard ratio (SHR) and 95% confidence interval (CI) for proximal aortic reoperations and as a hazard ratio (HR) and 95%CI for mortality after 10 years. Two-sided *p* < 0.05 was considered statistically significant. Statistical analyses were performed with the SPSS (version 29.0, SPSS Inc., IBM, Chicago, IL, USA) and Stata (version 15.1, StataCorp LLC, College Station, TX, USA) statistical software.

## 3. Results

**Figure 1:**
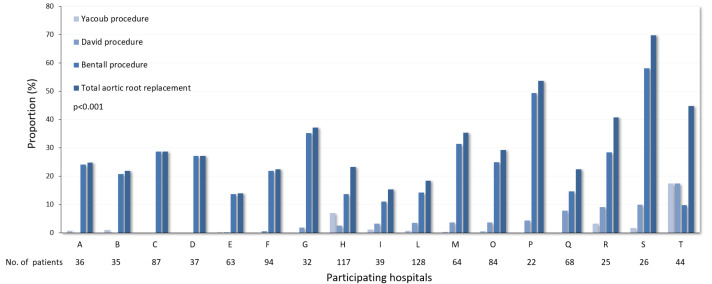
Proportions of different aortic root replacement procedures in the participating hospitals (*p* < 0.001 for all comparisons).

The ERTAAD dataset included data from 3735 consecutive patients who required surgery for acute TAAD. Aortic root replacement was performed in 1068 (28.6%) patients, consisting of a Bentall procedure in 862 (23.1%), a David procedure in 139 (3.7%), and a Yacoub procedure in 67 (1.8%). The latter patients were excluded from further analyses. The proportion of aortic root replacement, as well as the different techniques of aortic root replacement, varied significantly between the participating hospitals (*p* < 0.001) (Figure 1). For the purpose of the present study, 1001 patients who required a Bentall or a David procedure were included in the analysis. The baseline characteristics and operative data for these patients are summarized in Table 1. In the unmatched cohort, patients who required a Bentall procedure were older than those in the David procedure group (58.8 ± 12.7 vs. 54.8 ± 13.4 years, SD 0.31). The rates of bicuspid aortic valve (10.8% vs. 3.6%, SD 0.28) and diabetes were higher in the Bentall study group (5.1% vs. 2.2%, SD 0.15, respectively), while genetic syndromes were more represented in the David procedure group (9.4% vs. 4.9%, SD 0.28). No preoperative differences were observed between the two groups in terms of previous cardiac surgery (3.0% vs. 2.8%, SD 0.04), iatrogenic dissection (0.7% vs. 1.4%, SD 0.06), and previous stroke (2.2% vs. 3.7%, SD 0.04). Cerebral (18% vs. 19.7%, SD 0.04), spinal (1.4% vs. 1.7%, SD 0.002), and renal malperfusion (6.5% vs. 7.3%, SD 0.03) and peripheral malperfusion (11.5% vs. 15% SD 0.10) were equally distributed between the study cohorts. Moderate-to-severe aortic valve regurgitation was significantly more frequent in the Bentall procedure group (73.1% vs. 51.8%, SD 0.45).

Operative variables are listed in Table 1. Salvage procedures (2.9% vs. 5%, SD 0.10) were more frequent in the Bentall procedure group, as was the frequency of coronary artery bypass grafting (18% vs. 7.9%, SD 0.30). The aortic cross-clamping time was similar between the Bentall procedure and the David procedure cohorts (170 ± 66 vs. 168 ± 62, SD 0.02), but the cardiopulmonary bypass time was significantly longer in the Bentall procedure group (256 ± 94 vs. 272 ± 100, SD 0.17). Postoperative outcomes are listed in Table 2.

Propensity score matching yielded 115 pairs of patients with a comparable distribution of baseline and operative variables. The cardiopulmonary bypass time was longer after the Bentall procedure compared to the David procedure (260 ± 95 vs. 278 ±100, SD −0.18) with comparable durations of myocardial ischemia (Table 1).

In the matched cohorts, no statistical differences were observed in terms of postoperative complications, except for the rate of dialysis, which was higher in patients who underwent the Bentall procedure (17.4% vs. 7.0%, *p*-value 0.016).

**Figure 2:**
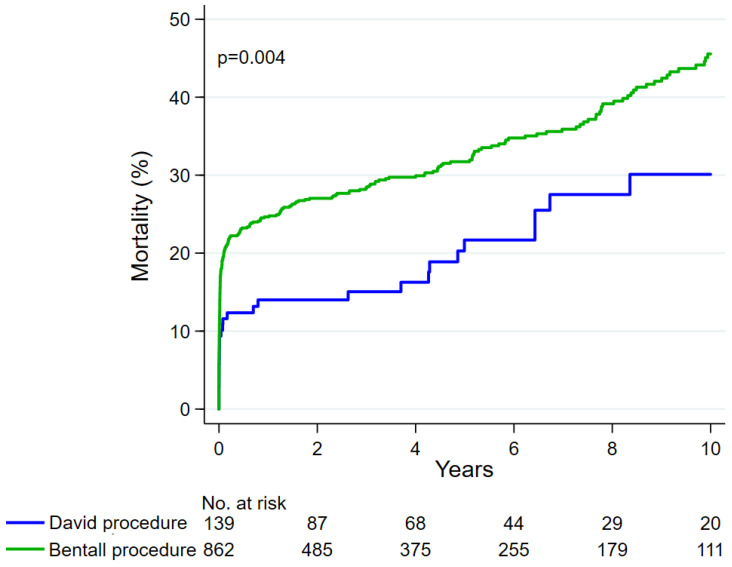
Mortality after the David procedure and the Bentall procedure in the overall series.

**Figure 3:**
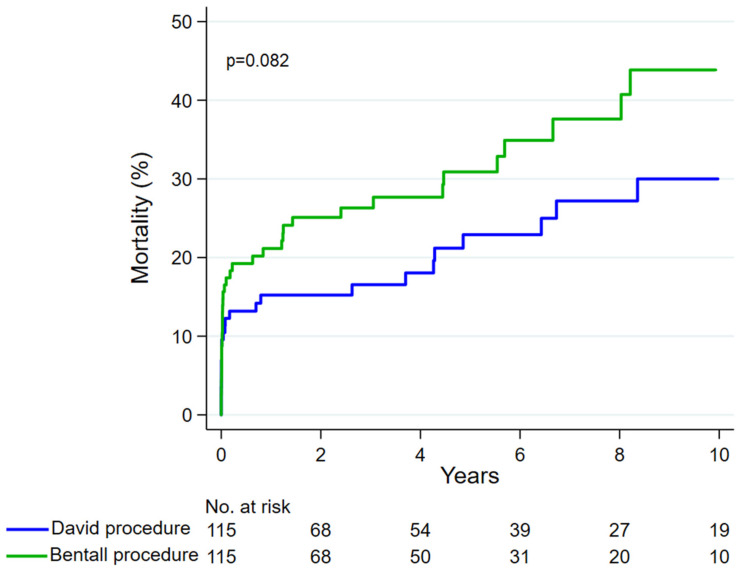
Mortality after the David procedure and the Bentall procedure among propensity score-matched patients.

In the unmatched cohorts, the David procedure was associated with a lower 10-year mortality rate compared to the Bentall procedure (30.1% vs. 45.6%, *p*-value 0.004) (Figure 2), but such a difference did not persist after propensity score matching (30.0% vs. 43.9%, *p*-value 0.082) (Figure 3). After 10 years, there were no differences in terms of proximal aortic reoperation (3.9% vs. 4.1%, *p*-value 0.954), even after matching (2.8% vs. 1.8%, *p*-value 0.994). The types of proximal aortic reoperation during the overall study period are summarized in Table 3. Among the propensity score-matched cohorts, over the entire study, proximal aortic reoperation was necessary in three patients after the David procedure (aortic valve repair; Bentall procedure and transcatheter aortic valve replacement; Bentall procedure twice) and in three patients after the Bentall procedure (local repair; local repair, Bentall procedure).

## 4. Discussion

In the present study, we did not observe any significant differences in terms of early postoperative complications (except for the higher incidence of dialysis in the Bentall group), long-term mortality, and proximal aortic reoperation after the Bentall and David procedures in patients operated on for acute TAAD.

In the case of TAAD involving the aortic root, aortic root replacement may be a durable procedure [7], but there are no recommendations about the surgical procedure of choice in this subset of patients. The present results confirm that both the Bentall and David procedures can be considered effective in the case of TAAD, with a low risk of late proximal aortic reoperation [8,9].

A previous study by Yang et al. [10] showed that the David procedure should be preferred to the Bentall procedure in young TAAD patients with stable hemodynamics and a favorable aortic valve anatomy. However, the study compared two cohorts of patients that were significantly different in terms of their baseline characteristics, which limited the generalizability of their findings.

A recent metanalysis by Mosbhi et al. [11], including 3058 patients from 27 retrospective studies, demonstrated that the David procedure provided better outcomes than the Bentall procedure in terms of aortic valve-related reintervention and survival. It is noteworthy that, in this meta-analysis, only one study in the David group reported a follow-up longer than 10 years.

The results of this study suggest that there are no differences in terms of the short- and long-term mortality rate between the Bentall and David procedures. However, the David procedure is technically more challenging compared to the Bentall procedure. Therefore, we may expect that, despite the balanced risk factors between the two study cohorts, the David procedure might have been performed by experienced surgeons with the aortic valve reimplantation technique and, more generally, with aortic surgery. Indeed, despite the complexity of the David procedure, this procedure was performed with a myocardial ischemia time comparable to that of the Bentall procedure. This finding suggests that, in experienced hands, the David procedure can be safely performed with the significant advantage of preserving the native aortic valve.

Chikwe et al. [12] suggested that relying on experienced surgeons in TAAD may be a strategy to reduce operative mortality and morbidity. Furthermore, the preoperative condition of the patient and the experience of the surgeon are considered the two main features for optimal outcomes of the operation [13].

A few limitations should be considered when evaluating the present results. First, the retrospective design of the ERTAAD is the main methodologic limitation. Second, the number of Bentall and David procedures performed in each center differed significantly. In fact, despite the robustness of the statistical analysis methods, few patients underwent the David procedure, suggesting that this procedure is only performed by a few experienced surgeons in a limited number of centers. Finally, the multicenter and retrospective nature of this study prevented an analysis of the criteria of eligibility for the David procedure adopted for these patients.

In conclusion, the David and Bentall procedures are durable treatment methods for TAAD. When feasible, it is advisable for the David procedure to be performed for acute TAAD by surgeons with experience with this demanding technique. Indeed, the David procedure allowed for the preservation of the native aortic valve with rates of postoperative complications, reintervention, and survival which were comparable to those for the Bentall procedure.

## Figures and Tables

**Table 1:** Baseline and operative variables in the unmatched and propensity score-matched cohorts.

	Unmatched Cohorts			Propensity Score-Matched Cohorts		
Baseline and Operative Variables	David Procedure N = 139	Bentall Procedure N = 862	Standardized Differences	David Procedure N = 115	Bentall Procedure N = 115	Standardized Differences
**Baseline variables**						
Age, years	54.8 (12.7)	58.8 (13.4)	−0.31	55.62 (12.7)	55 (14.6)	0.04
Females	23 (16.5)	182 (21.1)	0.12	20 (17.4)	19 (16.5)	0.02
eGFR, mL/min 1.73 m^2^	75 (26)	73 (23)	0.10	73 (26)	76 (25)	−0.12
Bicuspid aortic valve	5 (3.6)	93 (10.8)	0.28	5 (4.3)	6 (5.2)	0.04
Genetic syndrome	13 (9.4)	42 (4.9)	0.17	6 (5.2)	9 (7.8)	0.10
Prior cardiac surgery	3 (2.2)	24 (2.8)	0.04	3 (2.6)	2 (2.7)	0.06
Iatrogenic dissection	1 (0.7)	12 (1.4)	0.06	0 (0)	0 (0)	0.0
Diabetes	3 (2.2)	44 (5.1)	0.15	3 (2.6)	2 (1.7)	0.6
Prior stroke	3 (2.2)	32 (3.7)	0.09	3 (2.6)	1 (0.9)	0.13
Pulmonary disease	7 (5)	72 (8.4)	0.13	6 (5.2)	6 (5.2)	0.0
Extracardiac arteriopathy	2 (1.4)	31 (3.6)	0.14	2 (1.7)	1 (0.9)	0.08
**Preoperative malperfusion**						
Cerebral malperfusion	25 (18)	170 (19.7)	0.04	21 (18.3)	20 (17.4)	0.02
Spinal malperfusion	2 (1.4)	15 (1.7)	0.02	2 (1.7)	1 (0.9)	0.08
Renal malperfusion	9 (6.5)	63 (7.3)	0.03	7 (6.1)	5 (4.3)	0.08
Mesenteric malperfusion	5 (3.6)	28 (3.2)	0.02	4 (3.5)	4 (3.5)	0.0
Peripheral malperfusion	16 (11.5)	129 (15)	0.10	12 (10.4)	13 (11.3)	0.03
Preoperative cardiac massage	4 (2.9)	43 (5)	0.10	4 (3.5)	5 (4.3)	0.04
Invasive mechanical ventilation	2 (1.4)	81 (9.4)	0.35	2 (1.7)	1 (0.9)	0.08
Moderate-to-severe aortic valve insufficiency	72 (51.8)	626 (73.1)	0.45	67 (58.3)	58 (50.8)	0.16
**Operative variables**						
Salvage procedure	4 (2.9)	43 (5)	0.10	4 (3.5)	5 (4.3)	0.04
Coronary artery bypass grafting	11 (7.9)	155 (18)	0.30	9 (7.8)	10 (8.7)	0.03
Partial/total aortic arch replacement	37 (26.6)	153 (17.7)	0.22	28 (24.3)	33 (28.7)	0.10
Aortic cross-clamping time, min	170 (65)	168 (62)	0.02	170 (65)	173 (77)	−0.05
Cardiopulmonary bypass time, min	256 (94)	272 (100)	−0.17	260 (95)	278 (100)	−0.18

Continuous values are reported as mean and standard deviation (in parentheses). Categorical variables are reported as counts and percentages (in parentheses). Abbreviations: eGFR = estimated glomerular filtration rate according to the CKD-EPI equation; SD = standard deviation.

**Table 2:** Early and late outcomes in the unmatched and propensity score-matched cohorts.

	Unmatched Cohorts			Propensity Score-Matched Cohorts		
Postoperative Outcomes	David Procedure N = 139	Bentall Procedure N = 862	*p*-Values	David Procedure N = 115	Bentall Procedure N = 115	*p*-Values
Early outcomes						
Hospital death	16 (11.5)	170 (19.7)	0.021	13 (11.3)	18 (15.7)	0.334
Any stroke or global brain ischemia	18 (12.9)	154 (17.9)	0.154	14 (12.2)	18 (15.7)	0.446
Any stroke	16 (11.5)	127 (14.7)	0.314	13 (11.3)	17 (14.8)	0.434
Global brain ischemia	4 (2.9)	39 (4.5)	0.5	3 (2.6)	3 (2.6)	1
Paraparesis or paraplegia	3 (2.2)	45 (5.2)	0.136	2 (1.7)	2 (1.7)	1
Tetraplegia or tetraparesis	0	0	-	0	0	-
Mesenteric ischemia	5 (3.6)	32 (3.7)	1	3 (2.6)	2 (1.7)	1
Sepsis	13 (9.4)	120 (13.9)	0.141	9 (7.8)	6 (5.2)	0.423
Dialysis	9 (6.5)	114 (13.2)	0.024	8 (7.0)	20 (17.4)	0.016
Reoperation for intrathoracic bleeding	22 (15.8)	153 (17.7)	0.580	19 (16.5)	13 (11.3)	0.253
Deep sternal wound infection/mediastinitis	0 (0)	28 (3.2)	0.024	0 (0)	2 (1.7)	0.498
Heart failure	15 (10.8)	153 (17.7)	0.042	10 (8.7)	16 (13.9)	0.212
Mechanical circulatory support	4 (2.9)	47 (5.5)	0.296	4 (3.5)	8 (7)	0.375
VA-ECMO	2 (1.4)	40 (4.6)	0.107	2 (1.7)	7 (6.1)	0.171
Surgery for intestinal complications	0 (0)	4 (0.5)	1	0	0	-
10-year outcomes						
Mortality	29 (30.1)	295 (45.6)	0.004	25 (30.0)	37 (43.9)	0.082
Proximal aortic reoperation	4 (3.9)	25 (4.1)	0.954	2 (2.8)	2 (1.8)	0.994

Continuous values are reported as mean and standard deviation (in parentheses). Categorical variables are reported as counts and percentages (in parentheses). Abbreviations: VA-ECMO = Venoarterial extracorporeal membrane oxygenation.

**Table 3:** Proximal aortic reoperations after Bentall or David procedure.

No.	Primary Procedure	No. of Reoperations	Types of Proximal Aortic Reoperation
1	Bentall procedure	4	Local repair twice, replacement of the ascending aortic prosthesis, Bentall procedure
2	Bentall procedure	2	Bentall procedure twice
3	Bentall procedure	1	Replacement of the ascending aortic prosthesis
4	Bentall procedure	1	Local repair
5	Bentall procedure	1	Local repair
6	Bentall procedure	1	Local repair
7	Bentall procedure	1	Local repair
8	Bentall procedure	1	Local repair
9	Bentall procedure	1	Local repair
10	Bentall procedure	1	Local repair
11	Bentall procedure	1	Local repair
12	Bentall procedure	1	Bentall procedure
13	Bentall procedure	1	Bentall procedure
14	Bentall procedure	1	Bentall procedure
15	Bentall procedure	1	Bentall procedure
16	Bentall procedure	1	Bentall procedure
17	Bentall procedure	1	Bentall procedure
18	Bentall procedure	1	Bentall procedure
19	Bentall procedure	1	Bentall procedure
20	Bentall procedure	1	Bentall procedure
21	Bentall procedure	1	Bentall procedure
22	Bentall procedure	1	Bentall procedure
23	Bentall procedure	1	Bentall procedure
24	Bentall procedure	1	Bentall procedure
25	Bentall procedure	1	Surgical aortic valve replacement
26	Bentall procedure	1	Surgical aortic valve replacement
27	David procedure	2	Bentall procedure, transcatheter aortic valve replacement
28	David procedure	1	Bentall procedure
29	David procedure	1	Surgical aortic valve replacement
30	David procedure	1	Aortic valve repair
31	David procedure	1	Local repair

## Data Availability

The data in this study are not publicly available due to privacy issues.
